# Factors influencing the implementation of workplace-based health interventions for non-communicable disease prevention: a scoping review

**DOI:** 10.3389/fpubh.2026.1750114

**Published:** 2026-02-26

**Authors:** Aliza K. C. Bhandari, Kaung Suu Lwin, Phuong The Nguyen, Zin Wai Htay, Drishti Shrestha, Junko Saito, Akiko Yaguchi-Saito, Erika Ota, Taichi Shimazu

**Affiliations:** 1 Graduate School of Public Health, St. Luke’s International University, Tokyo, Japan; 2 School of Human and Health Sciences, University of Huddersfield, Huddersfield, United Kingdom; 3 Hitotsubashi Institute for Advanced Study (HIAS), Hitotsubashi University, Tokyo, Japan; 4 Department of Population Data Science, National Cancer Center Institute for Cancer Control, Tokyo, Japan; 5 Department of Global Health Policy, Graduate School of Medicine, The University of Tokyo, Tokyo, Japan; 6 Division of Behavioral Sciences, National Cancer Center Institute for Cancer Control, National Cancer Center, Tokyo, Japan; 7 Faculty of Human Sciences, Tokiwa University, Ibaraki, Japan; 8 Department of Global Health Nursing, Graduate School of Nursing Science, St. Luke’s International University, Tokyo, Japan

**Keywords:** barriers, facilitators, factors, health promotion interventions, implementation outcome, workplace

## Abstract

**Background:**

Workplace health promotion interventions are effective in improving employee health. However, most interventions are temporary and cannot be sustained, and a comprehensive understanding of the factors that influence implementation is limited. Therefore, using the implementation framework, this scoping review aims to identify the barriers and facilitators influencing the implementation of workplace health promotion interventions for the prevention of non-communicable diseases.

**Methods:**

We searched databases such as PubMed, Web of Science, and Scopus from January 1986 to August 2022 according to the Preferred Reporting Items for Systematic Reviews and Meta-Analyses (PRISMA) guidelines. Consistent with the Arksey and O’Malley framework, two independent reviewers reviewed the titles and abstracts for eligibility, followed by full-text screening using a data extraction form. Subsequently, a narrative summary of the barriers and facilitators identified from the included articles was synthesized and categorized into the Consolidated Framework for Implementation Research (CFIR). The identified barriers and facilitators were stratified according to the implementation outcomes.

**Results:**

Of the 38,384 articles identified, 610 articles were eligible for full-text screening, and 53 articles were included in the final analysis. Over 80% of the studies had applied a qualitative or mixed-methods approach, and the most common topics of intervention were reducing physical inactivity and promoting exercise (36%); moreover, 60% of the studies targeted interventions in medium- to large-sized organizations. Most factors were identified in the inner setting domain of the CFIR. The predominant facilitators were evidence strength and quality and leadership engagement, whereas structural characteristics, relative priority, and available resources were the most identified predominant barriers to the implementation of workplace health promotion interventions.

**Conclusion:**

The barriers and facilitators identified in this study can be used to implement a process to develop a strategy that targets the identified determinants to improve workplace health promotion interventions and their implementation.

## 1 Background

Noncommunicable diseases (NCDs), namely cardiovascular diseases, cancer, diabetes, and chronic lung disease, cause nearly three-fourths of annual deaths worldwide (1). The World Health Organization (WHO) and other global organizations support Target 3.4 of the Sustainable Development Goals (SDGs) that aim to reduce premature mortality caused by NCDs to one-third by 2030 (2). The WHO has extended its Global Action Plan 2013–2020 by another ten years to accelerate progress in the prevention and control of NCDs (3).

The WHO has highlighted the importance of the workplace in maintaining and promoting the health of individuals in the Third World Health Assembly in 1980 (4). Thus, the workplace is a valuable setting for implementing NCD prevention interventions as it can reach working adults for prolonged periods. According to the World Bank, the global labor force reached approximately 3.46 billion (nearly 44% of the world population) in 2021 (5). Based on this increment, it can be assumed that employees spend a lot of time at the workplace, and the amount of time spent working has gradually increased worldwide, including in the United States (US) (6). Furthermore, several work-related factors, such as prolonged working hours (7, 8), lack of motivation (9), sedentary work styles (10), overtime at work (11), and an unsanitary work environment (12), are associated with NCDs, including cancer, cardiovascular diseases, diabetes, and behavioral risk factors for chronic diseases. It is estimated that the global economic burden from NCDs over the period 2011–2030 will be approximately half of the gross domestic product in 2010 (13), and this is likely to increase the burden on employers because most of the NCDs are financed by the employee health insurance system (14). Thus, targeting workplaces could contribute toward population-wide reductions in preventing NCDs.

Evidence shows that workplace health promotion interventions (WHPIs) are effective in improving employee health (15–17). Several organizations have introduced various WHPIs; however, these interventions are mostly provided by various large-sized organizations. According to a 2017 survey conducted by the Center for Disease Control and Prevention in the US, nearly 92% of the worksites with more than 500 employees provided at least some form of worksite health promotion activity for their employees in 2017; however, the rate declined with a decrease in worksite size (18). The sustainable implementation of these WHPIs requires strong determination from employers along with resources and thoughtful processes from planning to the evaluation of desired outcomes (19). Many interventions are temporary or cannot be sustained owing to a lack of commitment from employers, poor intervention design, or employees’ unwillingness to participate in the WHPIs (20–22). Understanding these influencing factors can provide valuable insights for identifying subsequent implementation strategies. Several reviews have examined these influencing factors comprehensively or in specific areas (23, 24); however, to the best of our knowledge, none of these studies have organized these factors according to an implementation framework. Furthermore, as the barriers and facilitators for implementing WHPI can differ by the worksite or organizational structure (25, 26), it is important to identify these factors across various levels.

The Consolidated Framework for Implementation Research (CFIR) provides a broad spectrum of implementation research across five different domains: intervention characteristics, outer setting, inner setting, personal characteristics, and intervention process (27). Although CFIR was originally developed within health services research, it is not limited to clinical settings and has been increasingly applied to organizational and workplace-based interventions to examine multilevel implementation determinants (28). The CFIR provides an in-depth expression of ideas and helps researchers collect information, analyze, and interpret the findings in terms of the effectiveness of their intervention (28). It can be applied before, during, and after the implementation of an intervention, identify appropriate ways for developing an intervention, implementing it, and helping gather information on the sustainability of the intervention (29). As CFIR has been widely used in implementation research, this scoping review aims to identify the barriers and facilitators of WHPIs for the prevention of NCDs worldwide using CFIR and identify the evidence gaps to make recommendations for future implementation research in promoting workplace health.

## 2 Methods

We followed the Preferred Reporting Items for Systematic Reviews and Meta-Analyses Extension for Scoping Review (PRISMA-ScR) checklist (Supplementary Table 1) and reported our findings according to the Arksey and O’Malley framework. The protocol for this scoping review has been published previously (PLOS ONE, https://doi.org/10.1371/journal.pone.0275887) (30).

### 2.1 Research questions

We propose the following research question: What are the barriers and facilitators that influenced the implementation of workplace health promotion activities targeting NCDs?

### 2.2 Relevant studies

All relevant studies (e.g., qualitative, quantitative, and mixed-methods studies) have been included for evaluating the barriers and facilitators to implementing WHPIs. The studies were selected based on the following criteria:

#### 2.2.1 Population

We included studies focusing on stakeholders’ perspectives on barriers and facilitators to implementing WHPIs in their respective workplaces. Stakeholders play a direct role in the implementation of WHPIs, including but not limited to employers and management personnel at workplaces. We excluded studies focusing only on diseased populations, such as those studies conducted among specific groups of populations with pre-identified risks or chronic conditions such as obesity, diabetes, and hypertension.

#### 2.2.2 Concept

In this scoping review, we considered health-promoting interventions performed at the workplace focusing on modifiable lifestyle-related NCD prevention to address diet, physical activity, weight control, and tobacco and alcohol use at the workplace (e.g., smoke-free policies at workplaces and workplace fitness programs). We limited our studies to those that focused only on interventions for NCDs. Interventions for mental health prevention and studies focusing on interventions for the prevention of other diseases or conditions, such as communicable diseases, neglected diseases, and injuries, were excluded. Although the importance of mental health interventions in the workplace is increasing, factors influencing interventions are believed to be different from other lifestyle-related NCDs, as the involvement of staff with more specialized knowledge is important.

#### 2.2.3 Outcomes

The major outcomes of interest are barriers and facilitators to WHPI interventions. However, we excluded studies that did not mention at least one of the following eight implementation outcomes as a consequence of these barriers and facilitators: acceptability, adoption, appropriateness, costs, feasibility, implementation (fidelity), penetration, and sustainability (31). Studies focusing exclusively on determinants of employees’ participation, without addressing implications for organizational implementation processes, were excluded. Based on the proportion of studies identifying the construct as a facilitator (Pf) or barrier (Pb), we termed the factors as predominant barriers (Pb > Pf) or predominant facilitators (Pf > Pb); however, if the relative difference between Pf and Pb was similar, we termed them as indistinguishable factors (32).

#### 2.2.4 Context

Industry types were classified based on the international standard industrial classification of all economic activities.

### 2.3 Selection of studies

Using our search strategy, we examined databases such as PubMed, Web of Science, and Scopus from January 1, 1986, to August 31, 2022, applying the PRISMA guidelines. The detailed search strategy is presented in Supplementary Data Sheet 1. The search period was extended than mentioned in the protocol as there was some gap between the publication of the protocol and the preparation of the manuscript. The identified articles were aggregated into Rayyan, the common software and duplicates were removed. Subsequently, two independent reviewers reviewed the titles and abstracts for eligibility. A third reviewer resolved conflicts that arose between the two independent reviewers, and the total number of included studies was finalized. Further manual searches and the references of the included articles were checked to obtain any additional relevant articles.

### 2.4 Charting the data

The following information was extracted from articles that were included in the full-text screening process using a standardized data collection form: authors’ names, year of publication, country of publication, study design, framework used, study objective, study population, details of the intervention (e.g., type, size, and setting of the intervention), implementation outcome reported, and the description of barriers and/or facilitators to WHPIs implementation. We then coded the extracted descriptions of barriers and/or facilitators using all 39 constructs comprising the CFIR applying deductive content analysis (33). For a preliminary assessment of any coding conflicts, the two independent reviewers extracted the relevant information from approximately 5% of the included articles and coded them. All the conflicts were resolved based on the original CFIR definitions of each construct (34).

### 2.5 Collating, summarizing, and reporting the results

A narrative summary of the barriers and facilitators obtained from the included articles was provided using a data extraction form. A table comprising the numbers and percentages of each factor was then prepared, where the denominator was the total number of studies that identified the facilitators and/or barriers. The barriers and facilitators were stratified according to the implementation outcomes.

### 2.6 Consultation

We consulted two public health nurses involved in the implementation of WHPIs to gain insights.

### 2.7 Ethical considerations

Ethical approval was waived as this study used findings from the literature.

## 3 Results

**Figure 1:**
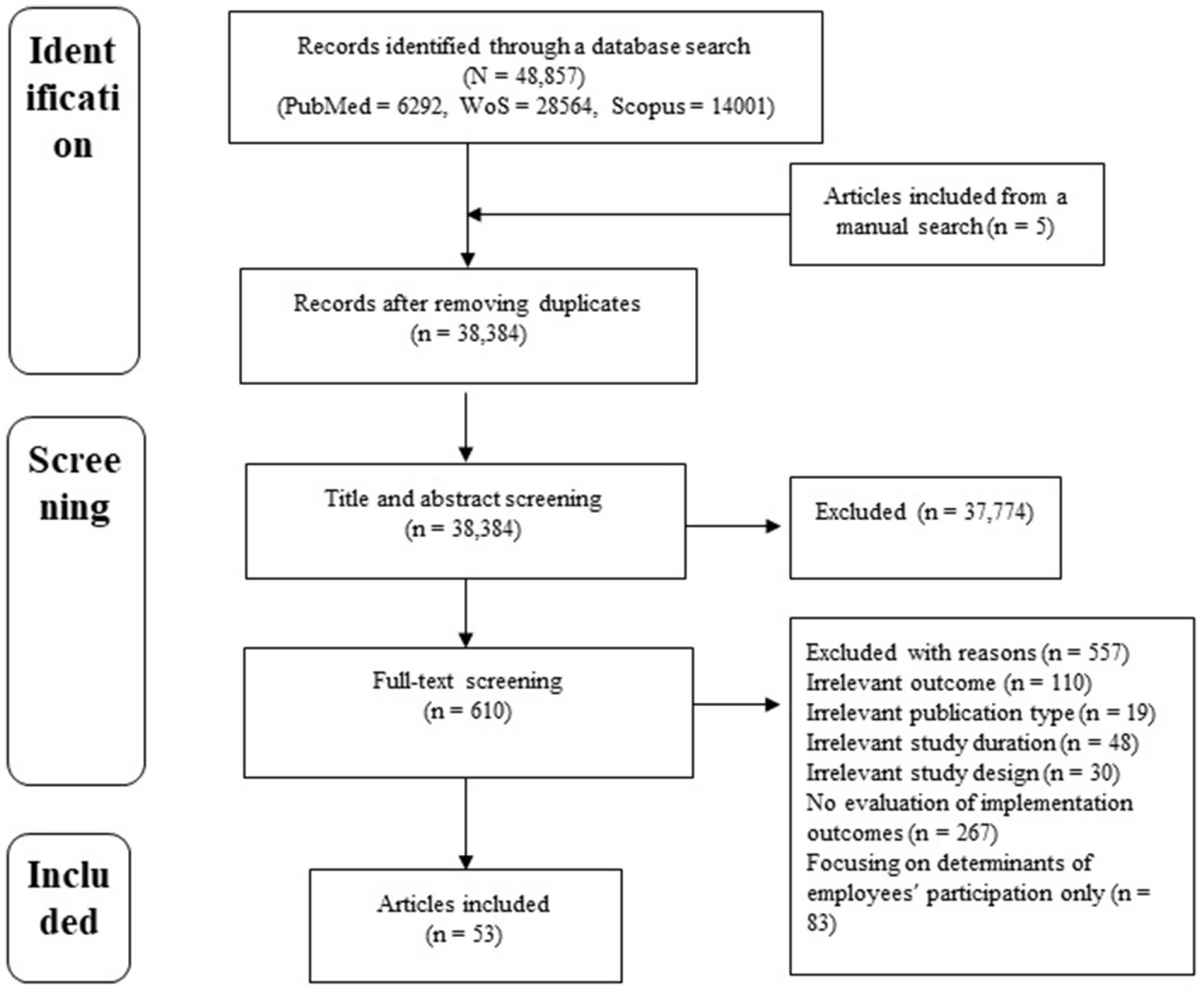
PRISMA flow diagram of the included articles.

A total of 38,384 articles were identified from PubMed, Scopus, and Web of Science after removing duplicates and then the selected articles were subjected to title and abstract screening. Only 610 articles were deemed eligible for full-text screening; however, 557 articles were excluded because of irrelevant information on outcomes, publication type, study duration, and study design. Some articles were also excluded because they were focusing exclusively on determinants of employees’ participation, without addressing implications for organizational implementation processes or there was no evaluation of the implementation outcomes. Hence, this scoping review reports a detailed synthesis of 53 articles (Figure 1).

### 3.1 Study characteristics

Table 1 describes the characteristics of the study sample. Approximately half (53%) of the included studies were qualitative studies (*N* = 28), followed by 15 mixed-methods (28%), and 10 quantitative (19%) studies. There has been an increase in the number of publications identifying the factors associated with the implementation of workplace interventions since 2015. Approximately 44% of the articles were from the US (*N* = 22), followed by countries in Europe (*N* = 13; 25%), Australia (*N* = 7; 13%), the United Kingdom (UK) (*N* = 4; 8%), and Asia, Africa, the Middle East, and Canada (*N* = 7; 13%). Detailed information on the included studies is provided in Supplementary Table 2.

**Table 1:** Information on studies included in this scoping review (*N* = 53).

Author (s)	Year of publication	Country	Study design	Framework (if any)	Industry type	Intervention	Size of workplace	Implementation outcome
Ablah et al. (37)	2019	US	Quan	The WorkWell Kansas Strategic Framework	–	Tobacco cessation, physical activity, and healthy eating	Large enterprises	Implementation
Adams et al. (53)	2017	UK	Quali	The RE-AIM Framework	N, P	Physical activity	Large enterprises	Adoption, implementation, penetration, and sustainability
Allen et al. (42)	2015	US	Quali	NA	I	Tobacco cessation, physical activity, and vaccination	Not specified	Adoption, implementation, and penetration
Bailey et al. (49)	2018	US	Quali	NA	–	Wellness	Micro, small, medium- and large-sized enterprises	Implementation
Banwell et al. (56)	2019	Australia	Quali	The Cultural Economy Framework	–	Physical activity, healthy eating strategies, and immunization	Small- and medium-sized enterprises	Adoption
Bramante et al. (83)	2017	US	Mm	NA	A, C, M, O, P, Q	Physical activity	Medium-sized enterprises	Adoption
Cameron et al. (84)	2018	Australia	Quali	NA	C	Alcohol cessation	Medium-sized enterprises	Adoption and sustainability
Coffeng et al. (50)	2013	Netherlands	Quan	The Framework of Steckler and Linnan	K	Physical activity	Medium-sized enterprises	Implementation
Crane et al. (48)	2019	Australia	Mm	NA	A, B, D, E, F, G, H, I, K, O, M, L, P, Q, R, S, T	Wellness	Micro, small, medium- and large-sized enterprises	Adoption
Danquah et al. (52)	2020	Denmark	Mm	The Nielsen & Randa Framework	–	Physical activity	Not specified	Implementation and penetration
Dubuy et al. (65)	2013	Belgium	Quali	The RE-AIM Framework	–	Exercise	Small- and medium-sized enterprises	Adoption, implementation, and sustainability
Elling et al. (85)	2020	Sweden	Quan	NA	F, H, K	Alcohol cessation	Medium- and large-sized enterprises	Adoption
Fitzgerald et al. (72)	2016	Ireland	Quali	The Steckler and Linnan Conceptual Framework	I, M, Q	Healthy eating strategies	Large enterprises	Implementation
Greenberg et al. (60)	2021	Israel	Mm	The RE-AIM Framework	O	Healthy eating strategies, physical activity, stress reduction, screening tests, smoking cessation, health awareness	Medium- and large-sized enterprises	Adoption, implementation, and sustainability
Hadgraft et al. (35)	2016	Australia	Quali	NA	G, M, Q	Physical activity	Small- and large-sized enterprises	Adoption, implementation, and penetration
Hannon et al. (38)	2012	US	Mm	NA	C, G, I, P, Q	Wellness	Medium- and large-sized enterprises	Adoption and implementation
Hannon et al. (36)	2012	US	Quali	NA	C, G, I, P, Q	Wellness	Medium- and large-sized enterprises	Adoption and implementation
Kava et al. (86)	2018	US	Quali	NA	C, G, F, K, L, O	Smoking cessation	Micro and small enterprises	Adoption and implementation
Kava et al. (87)	2022	US	Quali	NA	C, P, Q	Smoking cessation	Small- and medium-sized enterprises	Adoption and implementation
Klasen et al. (40)	2021	Netherlands	Quali	NA	B, C, K, P	Screening	Large enterprises	Adoption and implementation
Laing et al. (88)	2012	US	Quali	NA	–	Physical activity, healthy eating strategies, smoking cessation	Small- and medium-sized enterprises	Implementation
Leonard et al. (89)	2022	US	Quan	NA	C, O, P, Q	Physical activity	Small- and medium-sized enterprises	Implementation
Li et al. (90)	2018	China	Mm	NA	Q	Healthy eating strategies	Medium-sized enterprises	Implementation and sustainability
Lidegaard et al. (39)	2021	Denmark	Quali	NA	C	Smoking cessation	Large enterprises	Adoption
Lier et al. (43)	2019	Germany	Quan	NA	C, I, R, O	Physical activity and wellness	Medium- and large-sized enterprises	Penetration
Linnan et al. (66)	2019	US	Quan	NA	A, B, C, E, G, H, I, J, K, L, M, N, O, P, Q, R, S	Wellness	Small-, medium-, and large-sized enterprises	Adoption and implementation
Mandal et al. (51)	2021	India	Mm	NA	–	Smoking cessation	Medium- and large-sized enterprises	Implementation
Martinsson et al. (41)	2016	Sweden	Quali	NA	K	Incentives	Medium- and large-sized enterprises	Adoption and implementation
Mastenbroek et al. (61)	2022	Germany	Quali	NA	K	Physical activity	Micro-, small-, and medium-sized enterprises	Adoption and implementation
McCardel et al. (44)	2021	US	Mm	The CFIR Framework	O	Physical activity and healthy eating strategies	Large enterprises	Adoption and implementation
McLellan et al. (54)	2015	US	Quan	NA	A, B, C, E, G, H, I, J, K, L, M, N, O, P, Q, R, S	Wellness	Micro-, small-, medium-, and large-sized enterprises	Implementation and sustainability
Mellor et al. (57)	2013	UK	Mm	NA	O	Wellness	Large enterprises	Adoption and implementation
Montini et al. (91)	2008	US	Quali	NA	I	Smoking cessation	Large enterprises	Adoption and implementation
Morris et al. (82)	2019	UK	Mm	NA	J	Physical activity	Large enterprises	Implementation and penetration
Nelson et al. (92)	2015	US	Quali	NA	C	Wellness	Small- and medium-sized enterprises	Adoption and implementation
Person et al. (81)	2010	US	Quali	NA	P	Wellness	Large enterprises	Penetration
Pitts et al. (63)	2016	US	Mm	NA	Q	Healthy eating strategies	Medium- and large-sized enterprises	Adoption and sustainability
Rantala et al. (47)	2021	Finland	Mm	NA	–	Healthy eating strategies and physical activity	Medium- and large-sized enterprises	Implementation
Sargent et al. (59)	2018	Australia	Quali	NA	C, F, G, H, I, K, L, P, Q, R	Healthy lifestyle behavior and healthy environment	Small- and medium-sized enterprises	Implementation and penetration
Schouw et al. (46)	2018	Africa	Quali	NA	D	Healthy eating strategies and wellness	Large enterprises	Adoption, implementation, and sustainability
Seaton et al. (67)	2017	Canada	Mm	NA	H	Physical activity, healthy eating strategies, and incentives	Not specified	Adoption, implementation, and sustainability
Sigblad et al. (58)	2020	Sweden	Quali	NA	F, G, Q, S	Physical activity	Medium- and large-sized enterprises	Adoption
Strickland et al. (93)	2015	US	Quali	NA	G, Q	Physical activity and healthy eating strategies	Large enterprises	Implementation and penetration
Strickland et al. (55)	2019	US	Mm	The Formal Evaluation Framework	G	Physical activity and healthy eating strategies	Medium-sized enterprises	Implementation
Taylor et al. (45)	2016	Australia	Quan	NA	O, S	Wellness	Micro-, small-, medium-, and large-sized enterprises	Adoption and implementation
Tenney et al. (94)	2021	US	Quan	NA	–	Wellness	Small- and large-sized enterprises	Adoption and implementation
Verweij et al. (62)	2012	Netherlands	Quali	NA	Q	Physical activity and healthy eating strategies	Large enterprises	Adoption and implementation
Vyth et al. (71)	2011	Netherlands	Quan	NA	I	Healthy eating strategies	Large enterprises	Adoption and sustainability
Warehime et al. (76)	2019	US	Quali	NA	G, K, M, Q	Wellness	Large enterprises	Adoption and implementation
Welch et al. (95)	2020	Australia	Mm	The RE-AIM Framework	O, S	Exercise	Medium-and large-sized enterprises	Adoption, implementation, and sustainability
Wipfli et al. (64)	2018	US	Quali	NA	C, O, J, K, P, Q	Tobacco cessation, physical activity, wellness	Medium- and large-sized enterprises	Adoption
Wyatt et al. (96)	2015	UK	Quali	NA	I, J	Wellness	Medium- and large-sized enterprises	Implementation
Zou et al. (97)	2019	China	Quali	NA	C	Smoking cessation	Large enterprises	Implementation

Quali, qualitative method; Quan, quantitative method; Mm, mixed method; −, not available; Industry type: A, agriculture, forestry, and fishing; B, mining and quarrying; C, manufacturing; D, electricity, gas, steam, and air conditioning supply; E, water supply, sewerage, waste management and remediation activities; F, construction; G, wholesale and retail trade, repair of motor vehicles and motorcycles; H, transportation and storage; I, accommodation and food service activities; J, information and communication; K, financial and insurance activities; L, real estate activities; M, professional, scientific, and technical activities; N, administrative and support service activities; O, public administration and defense, compulsory social security; P, education; Q, human health and social work activities; R, arts, entertainment, and recreation; S, other service activities.

### 3.2 Characteristics of interventions and implementation outcomes

Approximately 36% of the studies focused their intervention on reducing physical inactivity and promoting exercise followed by wellness interventions (25%), healthy eating behaviors (13%), smoking cessation (11%), alcohol and tobacco control (7.5%), and others. However, approximately 15 studies (28%) focused on multiple interventions. Most interventions were implemented in medium- to large-scale organizations (64%). Only a limited number of studies utilized implementation frameworks such as RE-AIM (Reach, Effectiveness, Adoption, Implementation, and Maintenance) or CFIR in their studies. Among the eight implementation outcomes, barriers and facilitators identified in the included studies could be mapped to only four outcomes (adoption, implementation, penetration, and sustainability). All the studies had one or more implementation outcomes with most focusing on the implementation of interventions (44%) followed by adoption (37%) (Table 1).

### 3.3 Barriers and facilitators to WHPI implementation across the CFIR domain

Table 2 shows the frequencies and proportions of barriers and facilitators to WHPI implementation across various CFIR domains and constructs. The detailed codes of the facilitators and barriers across the five domains and 39 constructs of the CFIR are provided in Supplementary Table 3.

**Table 2:** Proportion of facilitators and barriers by CFIR domains and constructs.

CFIR domains and constructs	Facilitators (*N* = 46), *n* (%)	Barriers (*N* = 47), *n* (%)
Intervention characteristics		
Intervention source	1 (2%)	–
Evidence strength and quality	9 (20%)	4 (9%)
Relative advantage	4 (9%)	1 (2%)
Adaptability	4 (9%)	2 (4%)
Trialability	1 (2%)	1 (2%)
Complexity	3 (7%)	8 (17%)
Design quality and packaging	5 (11%)	4 (9%)
Cost	5 (11%)	9 (19%)
Outer setting		
Employees’ needs and resources	3 (7%)	7 (15%)
Cosmopolitanism	1 (2%)	–
Peer pressure	1 (2%)	2 (4%)
External policy and incentives	7 (15%)	1 (2%)
Inner setting		
Structural characteristics	3 (7%)	12 (26%)
Networks and communications	6 (13%)	7 (15%)
Culture	5 (11%)	9 (19%)
Implementation climate	2 (4%)	1 (2%)
Tension for change	2 (4%)	1 (2%)
Compatibility	6 (13%)	3 (6%)
Relative priority	–	12 (26%)
Organizational incentives and rewards	1 (2%)	–
Goals and feedback	2 (4%)	–
Learning climate	1 (2%)	–
Readiness for implementation	3 (7%)	2 (4%)
Leadership engagement	18 (39%)	14 (30%)
Available resources	9 (20%)	19 (40%)
Access to knowledge and information	6 (13%)	4 (9%)
Characteristics of individuals		
Knowledge and beliefs about the intervention	5 (11%)	6 (13%)
Self-efficacy	–	1 (2%)
Individual stage of change	2 (4%)	2 (4%)
Individual identification with organization	1 (2%)	2 (4%)
Other personal attributes	3 (7%)	1 (2%)
Process		
Planning	2 (4%)	2 (4%)
Engaging	7 (15%)	2 (4%)
Opinion leaders	–	–
Formally appointed internal implementation leaders	4 (9%)	2 (4%)
Champions	3 (7%)	–
External change agents	5 (11%)	1 (2%)
Executing	–	1 (2%)
Reflecting and evaluating	–	–

*N*, total number of studies identifying facilitators and barriers in workplace health interventions; *n*, number of times CFIR constructs were reported in studies as barriers or facilitators; “–” No study reported identifying the construct as a barrier or facilitator.

#### 3.3.1 Intervention characteristics

Some constructs, such as evidence strength and quality of the intervention (20%), relative advantage (9%), and adaptability (9%), were identified as the predominant facilitators of WHPI implementation, whereas complexity of the intervention (Pf = 7% and Pb = 17%) and cost (Pf = 11% and Pb = 19%) were identified as the predominant barriers to implementation. Other constructs, such as the intervention source, trialability, and design quality and packaging were identified as indistinguishable factors in the implementation of WHPI implementation (Pf – Pb ≥ 2%) (Table 2).

#### 3.3.2 Outer setting

External policy and incentives (15%) were identified as predominant facilitators and employees’ needs and resources (Pf = 7% and Pb = 15%) as predominant barriers, whereas cosmopolitanism and peer pressure were identified as indistinguishable factors in WHPI implementation (Table 2).

#### 3.3.3 Inner setting

The barriers and facilitators reported in the studies included in this review were in the inner setting of the CFIR domain. However, the identified factors were indistinguishable as barriers and facilitators in the constructs in the inner setting, such as networks and communication, implementation climate, tension for change, organizational incentives and rewards, and readiness for implementation. Compatibility (13%), goals and feedback (4%), leadership engagement (39%), and access to knowledge and information (13%) were identified as the predominant facilitators, whereas structural characteristics (26%), culture (19%), relative priority (26%), and available resources (40%) were identified as the predominant barriers. No study has yet identified relative priority as a facilitator, whereas leadership engagement was the most identified factor among all (*N* = 32) (Table 2).

#### 3.3.4 Characteristics of individuals

Few studies reported factors related to the characteristics of individuals as barriers or facilitators; moreover, studies that reported them did not provide a clear demarcation on either. These factors were the predominant facilitators or barriers. We identified other personal attributes (7%) as the predominant facilitators in this domain, while the rest were indistinguishable (Table 2).

#### 3.3.5 Process

Few studies focused on factors related to the process of WHPI implementation. No construct in this domain was identified as a predominant barrier to implementation; however, engaging (*n* = 7; 15%), stakeholders’ engagement (*n* = 2; 4%), formally appointed internal opinion leaders (*n* = 4; 9%), champions (*n* = 3; 7%), and external change agents (*n* = 5; 11%) were identified as the predominant facilitators, while the rest were indistinguishable (Table 2).

### 3.4 Predominant barriers and facilitators according to CFIR constructs

Very few CFIR constructs (five out of 39) were reported as the predominant facilitators or barriers by ≥ 20% of the studies (Table 2). The detailed findings of these five constructs are given in the following subsections. Supplementary Table 3 provides corresponding examples of the factors (barriers and facilitators).

#### 3.4.1 Evidence strength and quality

Among all other constructs in the intervention characteristics domain of the CFIR, evidence strength and quality were the only constructs with the strongest evidence, as more than 20% of the articles reported these constructs as facilitators (*N* = 9), whereas four studies (9%) identified them as barriers owing to a perceived lack of evidence in implementing certain interventions (35, 36). Hence, it was the predominant facilitator for implementing the intervention. Evidence to decrease the cost of intervention (37) and its perceived benefits to employees (38–40) facilitated the implementation of respective interventions. For example, in a focus group discussion, one of the participants mentioned, “*What works as an incentive is if you know that other workplaces that have used a workplace health intervention and have achieved results. This is something that works* (41).”

#### 3.4.2 Structural characteristics

Approximately 26% of the studies (*N* = 12) identified the structural characteristics of the organization such as a high employee turnover rate (42), size of the organization (43–45), and limited ability to reach all workers (36) as barriers to the implementation of WHPI interventions. Three studies (7%) reported this construct as a facilitator owing to the diversity of team members (46) and the presence of a support system within the organization (47). Hence, we considered structural characteristics as the predominant barriers to implementation.

#### 3.4.3 Relative priority

Relative priority was another predominant barrier (*N* = 12, 26%) identified within the inner setting with no studies reporting it as a facilitator. Conflicting priorities (48), difficulty in prioritizing the intervention over other organizational responsibilities (49, 50), and insufficient time provided to participate due to other responsibilities (51) were the specific barriers identified and classified in this construct.

#### 3.4.4 Leadership engagement

Leadership engagement was one of the most frequently identified factors among all other CFIR constructs (*N* = 32). Approximately 30% of the studies (*N* = 14) identified it as a barrier and approximately 39% (*N* = 18) as a facilitator. Hence, it was deemed a predominant WHPI implementation facilitator. For example, in a focus group discussion, one of the managers mentioned, “*We support it and say that you can do it in your working hours, including spending time preparing for it and stuff like that. That’s needed, of course* (52).” Barriers to leadership engagement include a lack of support from leaders (50, 53–55) and uncertainty of leaders’ commitment (38), whereas facilitators included support from leaders (52, 56), capacity of leaders to support implementation (38), leadership commitment (57), and positive attitudes (58).

#### 3.4.5 Available resources

The availability of resources was the most frequently identified construct among the barriers, as 40% of the studies (*N* = 19) identified it as a barrier and only 20% (*N* = 9) identified it as a facilitator. Therefore, this was a predominant barrier. In an interview, one of the managers highlighted, “*We probably would not be able to do it without the grant, especially given the financial constraints in that sector* (59).” Resources such as finance (60–62) working population, or employees (63) were considered as facilitators, whereas insufficient funding and finance (45, 53, 64), time constraints (56, 65) and labor shortage (66, 67) were considered barriers.

### 3.5 Barriers and facilitators according to implementation outcomes

Table 3 shows the facilitators and barriers of each CFIR construct according to implementation outcomes. The most frequently applied implementation outcomes for the identified facilitators and barriers were implementation (*N* = 213), adoption (*N* = 181), sustainability (*N* = 51), and penetration (*N* = 42). Other implementation outcomes (e.g., acceptability, appropriateness, and costs) are not presented in the table as we focused on the facilitators and barriers to implementation. Leadership engagement was the most frequently identified facilitator of adoption (6%) and implementation (6%), whereas it was the most frequently identified barrier to penetration (10%). The availability of resources was the most frequently identified facilitator of sustainability (8%) and the most frequently identified barrier to implementation (6%) and sustainability (10%). Regarding penetration, networks and communications were the most frequently identified facilitators (7%), whereas cost (10%) and leadership engagement (10%) were the most frequently identified barriers, followed by available resources (7%) (Table 3).

**Table 3:** Proportion of facilitators and barriers across CFIR constructs by four most cited implementation outcomes.

CFIR domains and constructs	Adoption (*N* = 181)		Implementation (*N* = 213)		Penetration (*N* = 42)		Sustainability (*N* = 51)	
	F, *n* (%)	B, *n* (%)	F, *n* (%)	B, *n* (%)	F, *n* (%)	B, *n* (%)	F, *n* (%)	B, *n* (%)
Intervention characteristics								
Intervention source	1 (1%)	–	–	–	–	–	–	–
Evidence strength and quality	6 (3%)	4 (2%)	6 (3%)	4 (2%)	1 (2%)	1 (2%)	1 (2%)	–
Relative advantage	3 (2%)	1 (1%)	2 (1%)	1 (0.5%)	–	–	1 (2%)	–
Adaptability	3 (2%)	2 (1%)	4 (2%)	1 (0.5%)	–	–	1 (2%)	1 (2%)
Trialability	–	1 (1%)	1 (0.5%)	–	–	–	–	–
Complexity	3 (2%)	5 (3%)	2 (1%)	4 (2%)	–	2 (5%)	1 (2%)	2 (4%)
Design quality and packaging	3 (2%)	2 (1%)	5 (2%)	2 (1%)	–	1 (2%)	1 (2%)	1 (2%)
Cost	3 (2%)	6 (3%)	3 (1%)	6 (3%)	2 (5%)	4 (10%)	–	4 (8%)
Outer setting								
Employees’ needs and resources	3 (2%)	6 (3%)	3 (1%)	6 (3%)	–	1 (2%)	–	–
Cosmopolitanism	1 (1%)	–	–	–	–	–	–	–
Peer pressure	1 (1%)	2 (1%)	1 (0.5%)	1 (0.5%)	–	–	–	–
External policies and incentives	5 (3%)	1 (1%)	5 (2%)	1 (0.5%)	–	–	–	–
Inner setting								
Structural characteristics	1 (1%)	8 (4%)	2 (1%)	10 (5%)	–	1 (2%)	1 (2%)	1 (2%)
Networks and communications	3 (2%)	3 (2%)	5 (2%)	5 (2%)	3 (7%)	–	1 (2%)	2 (4%)
Culture	3 (2%)	6 (3%)	5 (2%)	7 (3%)	–	1 (2%)	–	1 (2%)
Implementation climate	2 (1%)	–	2 (1%)	1 (0.5%)	–	1 (2%)	1 (2%)	–
Tension for change	1 (1%)	–	1 (0.5%)	1 (0.5%)	–	–	1 (2%)	–
Compatibility	4 (2%)	2 (1%)	3 (1%)	3 (1%)	–	1 (2%)	1 (2%)	–
Relative priority	–	7 (4%)	–	9 (4%)	–	2 (5%)	–	2 (4%)
Organizational incentives and rewards	–	–	1 (0.5%)	–	1 (2%)	–	–	–
Goals and feedback	2 (1%)	–	2 (1%)	–	–	–	1 (2%)	–
Learning climate	–	–	1 (0.5%)	–	–	–	–	–
Readiness for implementation	2 (1%)	1 (1%)	2 (1%)	2 (1%)	–	1 (2%)	–	–
Leadership engagement	11 (6%)	7 (4%)	13 (6%)	13 (6%)	3 (7%)	4 (10%)	2 (4%)	3 (6%)
Available resources	7 (4%)	12 (7%)	7 (3%)	13 (6%)	1 (2%)	3 (7%)	5 (10%)	5 (10%)
Access to knowledge and information	4 (2%)	2 (1%)	5 (2%)	3 (1%)	2 (5%)	1 (2%)	3 (6%)	–
Characteristics of individuals								
Knowledge and beliefs about the intervention	4 (2%)	5 (3%)	2 (1%)	3 (1%)	–	–	1 (2%)	1 (2%)
Self-efficacy	–	–	–	–	–	–	–	–
Individual stage of change	1 (1%)	1 (1%)	2 (1%)	1 (0.5%)	–	1 (2%)	1 (2%)	–
Individual identification with organization	1 (1%)	–	1 (0.5%)	2 (1%)	–	–	–	–
Other personal attributes	–	–	3 (1%)	1 (0.5%)	1 (2%)	–	–	–
Process								
Planning	1 (1%)	1 (1%)	2 (1%)	2 (1%)	–	–	1 (2%)	–
Engaging	5 (3%)	1 (1%)	6 (3%)	1 (0.5%)	–	–	3 (6%)	–
Opinion leaders	–	–	–	–	–	–	–	–
Formally appointed internal implementation leaders	1 (1%)	1 (1%)	4 (2%)	1 (0.5%)	–	1 (2%)	–	–
Champions	2 (1%)	–	1 (0.5%)	–	–	–	–	–
External change agents	4 (2%)	1 (1%)	4 (2%)	1 (0.5%)	–	–	1 (2%)	1 (2%)
Executing	–	1 (1%)	–	1 (0.5%)	–	–	–	–
Reflecting and evaluating	–	–	–	–	–	–	–	–

*N*, number of times CFIR constructs were identified in the respective implementation outcome; *n*, number of times CFIR constructs were identified as a facilitator or a barrier; F, facilitators; B, barriers; “–” No study reported identifying the construct as a barrier or facilitator.

### 3.6 Consultations

We consulted two public health nurses involved in WHPI to validate the study findings. They agreed that the predominant barriers and facilitators were reasonable from the perspective of real-world situations. In addition, the nurses reported that “formally appointed internal implementation leaders” in the process domain, which did not appear frequently in this review, could be highly influential factors because the presence and enthusiasm of the person in charge often alters the nature of health promotion activities in SMEs.

## 4 Discussion

To the best of our knowledge, this is the first study that focused on identifying the barriers and facilitators influencing WHPI implementation using the CFIR. This framework organizes information in common terms and summarizes findings from various studies using similar terminologies. Most studies identified the factors in the inner setting of the CFIR domain followed by intervention characteristics. In this study, we identified the predominant facilitators, barriers, and some indistinguishable factors affecting the implementation of WHPI activities.

### 4.1 Predominant facilitators

Evidence strength and quality of an intervention, and leadership engagement were the most frequently identified facilitators in this review, consistent with the findings of some other studies. Interventions proven to be effective were often perceived as suitable by employees (68). Such interventions not only increase employees’ interest in participation but also reduce the risk of NCDs in a given population (69). By contrast, poor-quality interventions lead to a lower participation rate and higher dropout rate, and this may also affect the sustainability of interventions in an organization. Leaders’ decision-making is central to adopting the program. However, continuous support after the adoption would also have a significant impact on enhancing WHPI implementation, as this could improve employee perception of the company’s commitment to implement health promotion practices, further improving employee well-being (70).

Similarly, interventions that had more advantages or were flexible were perceived as having better implementation outcomes (71, 72). Interventions that consider the needs of employees and are open to changes or amendments might improve the participation rates. Likewise, providing some forms of incentives is associated with an increase in employee job satisfaction and performance, also consistent with our findings (73). An organization’s readiness to implement WHPI interventions by providing sufficient information, knowledge, engagement, and leadership support can also enhance the implementation. This is consistent with the findings of previous studies that identified better job performance and work behavior among employees where leadership engagement was optimal (74, 75). Engaging stakeholders, leaders, or champions in the implementation process were identified as the predominant facilitators in this review, consistent with other studies (22). In the feedback from Japanese public health nurses, the inclusion of a formally appointed implementation leader among those involved in the WHPI was identified as a strong influencing factor and key to successful implementation. This scoping review identified this factor as a predominant facilitator as well. A possible attributable reason may be the different systems in which health programs are provided for example: in the US, vendors provide programs in general, whereas in several smaller companies in Japan, general affairs and human resources staff serve in tandem.

### 4.2 Predominant barriers

Lack of awareness of employees’ needs and resources and the inability to meet them owing to the organizational structure can be deemed as one of the biggest barriers to WHPI implementation (58, 76), consistent with some previous findings (77). Moreover, consistent with previous studies, having other priorities during the intervention also hindered implementation (78). The unavailability of resources such as money, time, and workforce were the most identified barriers to adoption, implementation, and sustainability. Several previous studies have supported this finding as a major barrier to the implementation of WHPI interventions (79). However, available resources were not the predominant facilitator of implementation. This indicates that the mere presence of resources is not sufficient for success because WHPI cannot be implemented without the engagement of leaders.

Complex and financially challenging interventions were identified as the predominant barriers in this scoping review, and this finding is consistent with previous studies (80). Securing funding, or a budget, and low-cost interventions enhance WHPI implementation; however, having insufficient funds or higher intervention costs could hamper the sustainability of an intervention. Hence, it is important to examine the cost-effectiveness and feasibility of an intervention before its implementation.

### 4.3 Indistinguishable factors

This scoping review has identified several indistinguishable factors; however, some key factors were considered as both facilitators and barriers in more than 10% of the included studies. Some studies identified having a well-designed (60) and sustainable interventions (72) as facilitators for implementation; however, others identified lengthy programs (81) and poor intervention designs (40) as barriers. Networks and communication are important components of implementation. However, whether it is a facilitator or a barrier depends on the situation. For example, one of the team leaders in a study stated, “*The only thing that I was doing was when the mails were coming through on a Monday, that’s when I would pick up with P13* (referring to a study participant) *so that would be the catalyst for the conversation with P13 to tell him or ask him how it’s going, that mail was a conversation starter for me to be fair*.” However, other team leaders mentioned, “*I think a lot of people would have looked at it* [recruitment email] *and thought more work if I* [am] *being honest with you* (82).” Hence, it is important for managers and program implementers to communicate appropriately with employees to enhance their active engagement. Similarly, having adequate knowledge and a good perception of the intervention enhances implementation, while inadequate knowledge could act as a barrier to implementation. Thus, it is important to provide knowledge about the intervention, outlining its benefits to employees’ health, so that they can perceive it well.

### 4.4 Strengths and limitations

This scoping review has identified the factors associated with WHPI implementation using the CFIR, and this has enabled us to present the results in a comprehensive and systematic manner. The strength of this review is the use of the CFIR, a comprehensive framework that provides a structure for understanding and analyzing contextual factors for implementation. The CFIR contributes to the general knowledge base on multi-level factors influencing WHP interventions across a diverse industry type. We also classified the factors by the implementation outcomes, and this has enabled us to specifically understand the knowledge gaps in the WHP context and facilitated further identification of strategies by the implementation phase, from pre- to post-implementation. However, this review has some limitations. First, while we searched three major interdisciplinary databases (PubMed, Scopus, and Web of Science) in line with the implementation-oriented scope of this review, we did not include specialized business databases such as Business Source. Second, the interventions identified in the included studies were diverse, and we were unable to identify the facilitators and barriers by the type of interventions. Third, the facilitators and barriers by industry type were not examined, although the industry types were diverse. The heterogeneity of interventions and industry types could limit generalizability as these variables may affect implementation differently. While CFIR offers a robust and widely used framework for capturing the comprehensive context in implementation science, it may not fully capture perspectives grounded in business or management science, which should be considered in future interdisciplinary research. Furthermore, in our review protocol, we reported our plan to extract health outcomes, if measured, and the changes in health outcomes after the intervention (i.e., whether they worsened, did not change, or improved after the implementation). However, as only a few studies reported the health outcomes of the target intervention, we had to deviate from the protocol and exclude the extraction of health outcomes.

## 5 Conclusion

This scoping review has identified and synthesized the barriers and facilitators to WHPI implementation using the CFIR, an implementation science framework. Most factors associated with WHPI implementation exist in the characteristics of the intervention and the inner setting. The predominant facilitators affecting WHPI implementation were evidence strength and quality (intervention characteristics), and leadership engagement (inner setting), whereas the predominant barriers were structural characteristics (inner setting), relative priority (inner setting), and available resources (inner setting). The next step in the implementation process is to develop a strategy that targets the identified determinants to improve WHPI implementation. Similarly, future reviews adopting an interdisciplinary scope may benefit from incorporating business-focused databases to provide a more comprehensive perspective on workplace-based health promotion interventions.

## Data Availability

The original contributions presented in the study are included in the article/Supplementary material, further inquiries can be directed to the corresponding author.
