# Supplementary material for: Factors influencing the implementation of workplace-based health interventions for non-communicable disease prevention: a scoping review
Source: Front Public Health. 2026 Feb 26;14:1750114. doi: 10.3389/fpubh.2026.1750114 (PMC12979459; doi:10.3389/fpubh.2026.1750114)
Supplement: Supplementary file 1 [file Data_Sheet_1.PDF]

Scopus search strategy

[Factors]

TITLE-ABS (barrier* OR challeng* OR limit* OR complicat* OR obstacle* OR hindrance* OR hinder* OR obstruct* OR restrain* or restrict* OR interfer* OR influen* OR imped* OR facilitat* OR enable* OR benefit* OR promot* OR empower* OR accept* OR factor* OR determinant*) AND

[Implementation]

({implementation science} OR implement* OR acceptab* OR satisfaction OR agreeability OR adoption OR uptake OR {knowledge translation} OR {intention to adapt} OR utilization OR appropriateness OR {perceived fit} OR relevance OR applicability OR compatibility OR fitness OR {cost-benefit analysis} or cost OR {cost-effect*} OR feasibility OR transferability OR practicability OR workability OR {actual fit} OR {actual utility} OR fidelity OR integrity OR {delivered as intended} OR {quality of program delivery} OR penetration OR {integration of practice} OR infiltration OR sustainability OR maintenance OR routinization OR durability OR institutionalization OR {capacity building} OR *continuation OR incorporation OR {sustained use} OR adhere OR participat*) AND

[Health promotion interventions]

(innovation OR EBP OR {evidence-based practice} OR {evidence based practice} OR {health promotion} OR {health management} OR {Health protection} OR {Well-being} OR obes* or {weight gain} or {weight loss} or overweight or {over-weight} or overeat* or {over eat} or {weight change*} or ((bmi or {body mass index}) and (gain or loss or change)) or {primary prevention} or {preventive measure*} or {preventative measure*} or {preventive care} or {preventative care} or (obesity and (prevent* or treat*)) or exercise or {physical inactivity} or {physical activity} or {Motor Activity} or ({physical education and training}) or {Physical Fitness} or sedentary or {Life Style} or {Leisure Activit*} or sport* or dancing or diet or nutrition* or {healthy eating} or fruit* or vegetable* or canteen or food or menu or calorie* or {energy intake} or {energy density} or eating or {feeding behavior} or {dietary intake} or {soft drink*} or soda or {sweetened drink*} or fat or confectionary or {feeding program*} or cafeteria* or ((smok* or tobacco or nictotine) and (cessation or stop* or quit* or abstin* or abstain* or reduc* or ex-smoker* or anti-smok*)) or ((alcohol* or drink* or liquor* or beer* or wine* or spirit* or drunk* or intoxicat* or binge))) AND

[workplace]

(job OR workplace* OR worksite* OR work site* OR {work place*} OR {at work} OR {job site*} OR occupation* OR employ*)AND DOCTYPE (ar) AND SRCTYPE (j) AND

LANGUAGE (english)

Notes: refined document type.
review or {meta analysis} or{news} or comments or editorial or {systematic review} or {literature review}

Number of retrieved articles: 14,001

*after removing duplicates and excluding the systematic reviews, meta-analysis, notes, comments, editorials, news, articles other than English language and articles before 1999 Jan 1.

Web of Science search strategy

[Factors]

TS=(barrier* OR challeng* OR limit* OR complicat* OR obstacle* OR hindrance* OR hinder* OR obstruct* OR restrain* or restrict* OR interfer* OR influen* OR imped* OR facilitat* OR enable* OR benefit* OR promot* OR empower* OR accept* OR factor* OR determinant*) AND

[Implementation]

TS=("implementation science" OR implement* OR acceptab* OR satisfaction OR agreeability OR adoption OR uptake OR "knowledge translation" OR "intention to adapt" OR utilization OR appropriateness OR "perceived fit" OR relevance OR applicability OR compatibility OR fitness OR "cost-benefit analysis" or cost OR "cost-effect*" OR feasibility OR transferability OR practicability OR workability OR "actual fit" OR "actual utility" OR fidelity OR integrity OR "delivered as intended" OR "quality of program delivery" OR penetration OR "integration of practice" OR infiltration OR sustainability OR maintenance OR routinization OR durability OR institutionalization OR "capacity building" OR *continuation OR incorporation OR "sustained use" OR adhere OR participat*) AND

[Health promotion interventions]

TS=(innovation OR EBP OR "evidence-based practice" OR "evidence based practice" OR "health promotion" OR "health management" OR "Health protection" OR "Well-being" OR obes* or "weight gain" or "weight loss" or overweight or "over-weight" or overeat* or "over eat" or "weight change*" or ((bmi or "body mass index") and (gain or loss or change)) or "primary prevention" or "preventive measure*" or "preventative measure*" or "preventive care" or "preventative care" or (obesity and (prevent* or treat*)) or exercise or "physical inactivity" or "physical activity" or "Motor Activity" or ("physical education and training") or "Physical Fitness" or sedentary or "Life Style" or "Leisure Activit*" or sport* or dancing or diet or nutrition* or "healthy eating" or fruit* or vegetable* or canteen or food or menu or calorie* or "energy intake" or "energy density" or eating or "feeding behavior" or "dietary intake" or "soft drink*"or soda or "sweetened drink*"or fat or confectionary or "feeding program*"or cafeteria* or ((smok* or tobacco or nictotine) and (cessation or stop* or quit* or abstin* or abstain* or reduc* or ex-smoker* or anti-smok*)) or (alcohol* or drink* or liquor* or beer* or wine* or spirit* or drunk* or intoxicat* or binge)) AND

[workplace]

TS=(job OR workplace* OR worksite* OR work site* OR "work place*" OR "at work" OR "job site*" OR occupation* OR employ*)

Notes: refined document type by year and language. review or {meta analysis} or{news} or comments or editorial or {systematic review} or {literature review}

Number of retrieved articles: 28,564

*after removing duplicates and excluding the systematic reviews, meta-analysis, notes, comments, editorials, news, articles other than English language and articles before 1999 Jan 1.

PubMed search strategy:

[Factors]

barrier* OR challeng* OR limit* OR complicat* OR obstacle* OR hindrance* OR hinder* OR obstruct* OR restrain* or restrict* OR interfer* OR influen* OR imped* OR facilitat* OR enable* OR benefit* OR promot* OR empower* OR accept* OR factor* OR determinant*

[Implementation]

“implementation science” OR implement* OR acceptab* OR satisfaction OR agreeability OR adoption OR uptake OR “knowledge translation” OR “intention to adapt” OR utilization OR appropriateness OR “perceived fit” OR relevance OR applicability OR compatibility OR fitness OR “cost-benefit analysis” or cost OR “cost-effect*” OR feasibility OR transferability OR practicability OR workability OR “actual fit” OR “actual utility” OR fidelity OR integrity OR “delivered as intended” OR “quality of program delivery” OR penetration OR “integration of practice” OR infiltration OR sustainability OR maintenance OR routinization OR durability OR institutionalization OR “capacity building” OR *continuation OR incorporation OR “sustained use” OR adhere OR participat*

[Health promotion interventions]

innovation OR EBP OR "evidence-based practice" OR “evidence based practice” OR "health promotion" OR "health management" OR "Health protection" OR “Well-being” OR obes* or "weight gain" or "weight loss" or overweight or "over-weight" or overeat* or "over eat" or "weight change*" or ((bmi or "body mass index") and (gain or loss or change)) or "primary prevention" or "preventive measure*" or "preventative measure*" or "preventive care" or "preventative care" or (obesity and (prevent* or treat*)) or exercise or "physical inactivity" or "physical activity" or "Motor Activity" or ("physical education and training") or "Physical Fitness" or sedentary or "Life Style" or "L eisure Activit*" or sport* or dancing or diet or nutrition* or "healthy eating" or fruit* or vegetable* or canteen or food or menu or calorie* or "energy intake" or "energy density" or eating or "feeding behavior" or "dietary intake" or "soft drink*"or soda or "sweetened drink*"or fat or confectionary or "feeding program*"or cafeteria* or ((smok* or tobacco or nictotine) and (cessation or stop* or quit* or abstin* or abstain* or reduc* or ex-smoker* or anti-smok*) or alcohol* or drink* or liquor* or beer* or wine* or spirit* or drunk* or intoxicat* or binge)

[workplace]

job OR workplace* OR worksite* OR work site* OR “work place*” OR “at work” OR “job site*” OR occupation* OR employ*

Number of retrieved articles: 6,292

*after removing duplicates and excluding the systematic reviews, meta-analysis, notes, comments, editorials, news, articles other than English language and articles before 1999 Jan 1.
